# Comparison of tolerability and adverse symptoms in oxcarbazepine and carbamazepine in the treatment of trigeminal neuralgia and neuralgiform headaches using the Liverpool Adverse Events Profile (AEP)

**DOI:** 10.1186/s10194-015-0563-z

**Published:** 2015-09-03

**Authors:** E. Besi, D. R. Boniface, R. Cregg, J. M. Zakrzewska

**Affiliations:** Facial Pain Unit, Eastman Dental Hospital, UCLH NHS Foundation Trust, 256 Gray′s Inn Road, London, WC1X 8LD UK; Department of Epidemiology, UCL, Health Behaviour Research Centre, 1-19 Torrington Place, London, WC1E 6BT UK; Pain Management Centre National Hospital for Neurology and Neurosurgery, 1st Floor Queen Mary Wing, Queen Square, London, WC1N 3BG UK

**Keywords:** Drug-related side effects and adverse reactions, Carbamazepine, Oxcarbazepine, Trigeminal neuralgia

## Abstract

**Background:**

Adverse effects of drugs are poorly reported in the literature . The aim of this study was to examine the frequency of the adverse events of antiepileptic drugs (AEDs), in particular carbamazepine (CBZ) and oxcarbazepine (OXC) in patients with neuralgiform pain using the psychometrically tested Liverpool Adverse Events Profile (AEP) and provide clinicians with guidance as to when to change management.

**Methods:**

The study was conducted as a clinical prospective observational exploratory survey of 161 patients with idiopathic trigeminal neuralgia and its variants of whom 79 were on montherapy who attended a specialist clinic in a London teaching hospital over a period of 2 years. At each consultation they completed the AEP questionnaire which provides scores of 19–76 with toxic levels being considered as scores >45.

**Results:**

The most common significant side effects were: tiredness 31.3 %, sleepiness 18.2 %, memory problems 22.7 %, disturbed sleep 14.1 %, difficulty concentrating and unsteadiness 11.6 %. Females reported significantly more side effects than males. Potential toxic dose for females is approximately 1200 mg of OXC and 800 mg of CBZ and1800mg of OXC and 1200 mg of CBZ for males.

**Conclusions:**

CBZ and OXC are associated with cognitive impairment. Pharmacokinetic and pharmacodynamic differences are likely to be the reason for gender differences in reporting side effects. Potentially, females need to be prescribed lower dosages in view of their tendency to reach toxic levels at lower dosages.

Side effects associated with AED could be a major reason for changing drugs or to consider a referral for surgical management.

## Background

Trigeminal neuralgia (TN) and closely related neuralgiform headache (NH) conditions such as short unilateral neuralgiform headache with autonomic symptoms (SUNA) are certain forms of neuropathic pain which are effectively managed by antiepileptic drugs (AED) such as carbamazepine (CBZ) and oxcarbazepine (OXC).

A recent study of Cochrane systematic reviews and their primary studies showed that specific harm outcomes are poorly reported and less than 30 % report them in full [1]. This is equally true in RCTs of AEDs in TN [2]. It is increasingly important to determine the overall and dose-dependent tolerability of these drugs as this is becoming a well recognized reason for change of therapy [3]. Patients with TN have reported experiencing a mean of three adverse effects when on AEDs and these are often underestimated by clinicians [4].

The aim of this study was to examine the frequency of the adverse events of AEDs, in particular CBZ and OXC in patients with neuralgiform pain using the Liverpool Adverse Events Profile (AEP) [5] a psychometrically tested self-complete questionnaire to determine whether these effects are dose and drug related. This would provide clinicians with guidance as to when to change management.

## Methods

The data were collected prospectively as part of routine care for all patients with idiopathic TN and SUNA attending a specialist clinic in a London teaching hospital over a period of 2 years. Patients were reviewed on average twice yearly unless they had stopped medications or were on a very low dose. Within this time period 161 patients were seen. Of these 19 were off all medications and therefore were excluded. 79 patients were on monotherapy of either CBZ or OXC, 63 were on either CBZ or OXC plus another anti neuropathic / analgesic medication which were included in the analysis. 54 out of the 161 patients were experiencing little or no pain due to the effective medical therapy instigated. Monotherapy was by default our initial approach in pharmacotherapy. All patients were initially prescribed CBZ and if they developed significant side effects they were changed to OXC. If patients had been prescribed other AEDs these were not changed till efficacy or tolerability was affected. Patients with SUNA were treated with OXC or lamotrigine either as single therapy or polytherapy. Two AEDs were used in those who had reached optimal levels of CBZ or OXC and were not candidates for surgery. All patients, as part of their routine follow up, complete a number of questionnaires and among them the AEP [6] in relation to the current drugs being taken for pain control. The Liverpool AEP [5] is a self-complete questionnaire consisting of 19 brief items :(disturbed sleep, memory problems, depression, sleepiness, dizziness, weight gain, shaky hands, trouble with mouth or gums, difficulty in concentrating, upset stomach, double or blurred vision, problems with skin, hair loss, headache, nervousness and/or aggression, feelings of aggression, restlessness, tiredness, unsteadiness) scored using a Likert scale of 1 to 4, thus achieving scores ranging from 19 to 76. Its psychometric properties have been found to be appropriate with an internal consistency of 0.95 and test-retest reliability of 0.85 [5]. It has been used in a European epilepsy study involving 5000 patients [7]. Scores over 45 suggest toxicity and should alert clinicans to consider a change of managment [8].

After completion in the waiting room the AEP was checked during the consultation by one clinician (JZ). This was done at each clinical visit. Efficacy was measured on the Brief Pain Inventory (BPI) and by the clinician global impression of change.

All patients were over 18 years of age and had sufficient cognitive function and English language skills to complete the questionnaires. Patients who were on drugs that may affect cognitive function e.g. psychiatric drugs including tricyclic antidepressants were excluded. All patients were weighed in their indoor clothing so their medication dosages could be converted to mg/kg. The total duration of drug use was recorded as well as the daily total drug dose used over the last two weeks. Hematological and biochemical tests were carried out initially two monthly but once stabilized on average on a yearly basis unless high doses were used when these were done three monthly. No hematopoietic abnormalities were found.

### Data analysis

The seventy nine patients with an average of 1.58 clinic attendances (range 1 to 4) who were on monotherapy consisting of either CBZ or OXC, and had completed the AEP questionnaire, were selected for analysis. Statistical analyses were carried out to determine the effect of drug and dose on adverse symptom profile and overall score. Analyses were carried out using multilevel statistical models to allow for the clustering of observations within patients. To take account of the differing dose of CBZ and OXC, for the same efficacy, doses were measured as multiples of the standard basic dose (SBD) of each drug. The basic dose was taken to be 200 mg for CBZ and 300 mg for OXC [9]. This resulted in doses ranging from 0.5 to 7.5 multiples of the SBD. All data analysis was performed with SPSS version 22 or Stata version 13.

## Results and discussion

Summary descriptive statistics of the seventy nine patients who were taking monotherapy consisting of either CBZ or OXC are shown in Table 1 which shows the distribution of age, weight, diagnoses, drug and sex according to the two factors of primary interest in the paper –i.e. sex and drug in the 79 patients involved in this analysis. Reassuringly, it shows remarkable uniformity in the distributions.

**Table 1:** Demographics, drugs, and diagnoses by sex and by drug

	Sex		Drug		
	Males (*n* = 36)	Females (*n* = 43)	CBZ (*n* = 26)	OXC (*n* = 49)	Changing drug (*n* = 4)
Age years (mean)	64.2	62.3	61.7	64.2	60.5
(range)	(40 to 89)	(29 to 92)	(35 to 82)	(29 to 92)	(47 to 67)
Weight kg (mean)	79.5	67.3	71.9	73.6	68.3
(95 % CI)	(75.5-83.5)	(63.7-70.9)	(67.5-76.3)	(69.4-77.8)	(60.4-76.1)
Diagnosis (%)					
TN	91.7	97.7	96.2	93.9	100.0
SUNA	8.3	2.3	3.9	6.1	0.0
Drug (%)					
CBZ	33.3	32.6	-	-	-
OXC	63.9	60.5	-	-	-
Changing drug	2.8	7.0	-	-	-
Males	-	-	46.2	46.9	25.0
Females	-	-	53.8	53.1	75.0

**Fig. 1:**
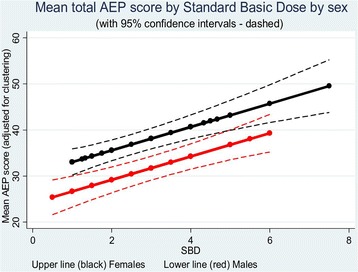
Linear multilevel models for total AEP score responses by SBD for males and females

Results of the analyses by multilevel linear and logistic regression models are set out in Table 2. Dose appears to be strongly linked to AEP total score after allowing for gender and drug (*p* < 0.001). Females report higher scores than males (*p* < 0.001). There is no evidence for difference in scores due to drug after efficacy, dose and gender are taken into account. The SBD coefficient from the regression model indicates that a single unit increase of SBD results in an increase of 1.98 units (95 % CI: 0.97 to 3.00) in the total AEP score (*p* < 0.001). In males as compared to females the total AEP score was some 6.65 points lower (95 % CI: 2.64 to 10.66) (*p* = 0.001). See Fig. 1. Taking CBZ rather than OXC resulted in total AEP score 2.71 points lower (95 % CI: 1.08 to 6.51) (*p* = 0.160). Adjustment of dose for differences in body weight had minimal impact on these results.

**Table 2:** Dependency of AEP total score and odds of exceeding a toxic score (>45) on SBD, gender and drug

	**Linear model coefficients** **†** **(** ***p*** **-values)**		
	SBD	Sex	Drug
AEP total score	1.98 (*p* < 0.001)	−6.65 (*p* = 0.001)	−2.71 (*p* = 0.160)
95 % CI	(0.97 to 3.00)	(−10.66 to −2.64)	(−6.51 to 1.08)
	**Logistic model Odds Ratios** **†** **(** ***p*** **-values)**		
	SBD	Sex	Drug
AEP total score >45	1.94 (*p* = 0.002)	0.111 (*p *= 0.003)	0.605 (*p* = 0.434)
95 % CI	(1.283 to 2.941)	(0.026 to 0.474)	(0.170 to 2.148)

†Interactions did not reach statistical significance and so were dropped from the models in order to improve clarity of results

A similar pattern was found for the odds that a patient would report a total AEP score above 45, Table 2.

**Fig. 2:**
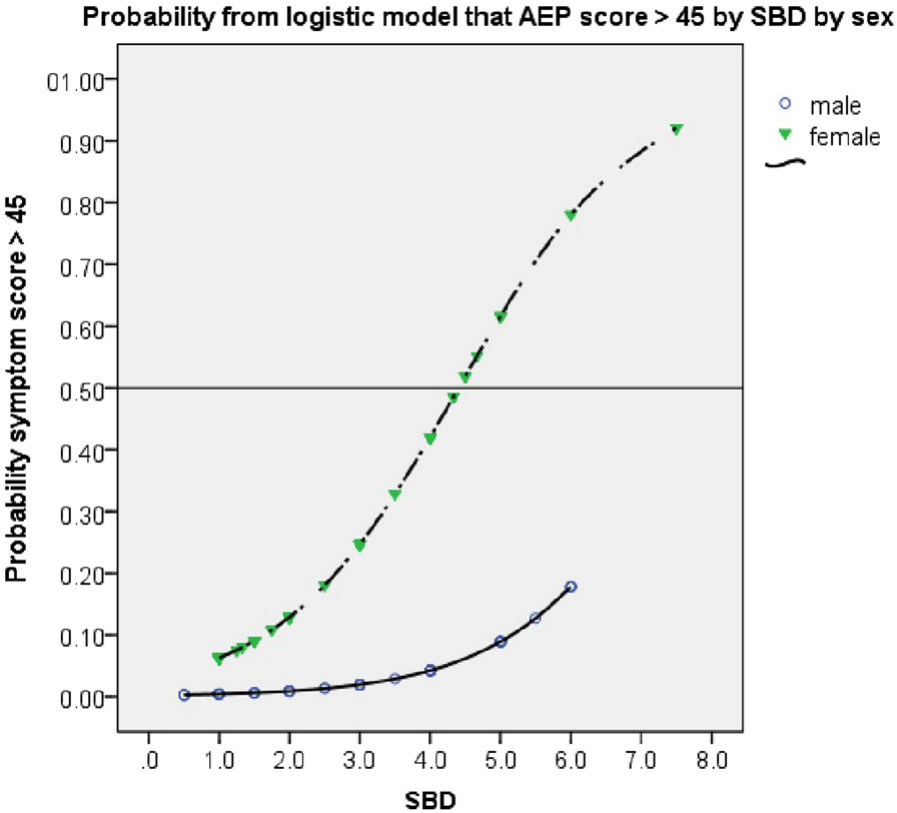
Probability from logistic model that AEP score exceeds 45 by SBD for males and females. The probability of a patient exceeding the threshold of 45 on the total symptom score is calculated from the fitted statistical model. This naturally follows the shape of a logistic curve as the model is fitting the log odds in a linear regression

According to the plotted curves for males and females in Fig 2 it is evident that in females a dose four times the standard basic dose corresponds to a 50 % probability of AEP score response greater than 45 (the toxic level).

For males it appears that the probability of exceeding the toxic level of AEP score is low even at six times the SBD. This difference between males and females is statistically significant (*p* < 0.001). Thus a 50 % probability of a toxic dose for females arises at around 1200 mg of OXC and at around 800 mg of CBZ. For males it is only 20 % likely at around 6.0 SBDs i.e. for a dose of 1800gm of OXC and 1200 mg of CBZ.

**Fig. 3:**
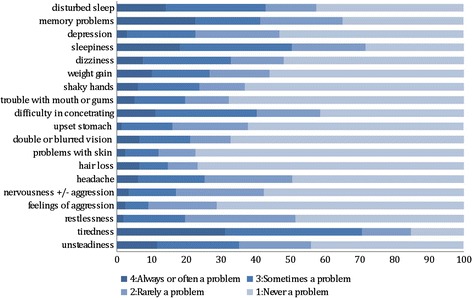
Side effects as reported on AEP questionnaire (%) in 79 patients on OXC and CBZ only. The 79 patients attended 125 times the outpatient clinic

Patients completed the AEP questionnaire at each visit and the most common side effects were found to be: tiredness 31.3 %, sleepiness 18.2 %, memory problems 22.7 %, disturbed sleep 14.1 %, difficulty concentrating and unsteadiness 11.6 % (Fig. 3).

Statistical analysis of these six most common side effects found that a one unit SBD increase results in around 50 % increase in odds of a one-step higher symptom response such as from level 1 to level 2, or from level 2 to level 3 on one of the 19 symptoms. Being female typically doubles odds of a one-step higher response but quadruples odds for memory problems, disturbed sleep and unsteadiness. Being on OXC rather than CBZ is neutral except that it at least triples the odds of a response of a one-step higher score for disturbed sleep and difficulty concentrating (Table 3).

**Table 3:** Odds ratios of higher level ordinal response due to unit change in SBD, sex or drug in the six most common side effects (95 % CI)

AEP Symptom	SBD (CI)	Sex (CI)	Drug (CI)
Tiredness	1.65 (1.26 to 2.18)***	2.10 (0.91 to 4.84)	0.76 (0.33 to 1.78)
Sleepiness	1.28 (0.96 to 1.69)	2.29 (0.84 to 6.25)	0.80 (0.30 to 2.17)
Memory Problems	1.58 (1.09 to 2.29)*	4.56 (1.03 to 20.27)*	1.55 (0.41 to 5.85)
Disturbed sleep	1.62 (1.07 to 2.44)*	4.31 (0.87 to 21.40)	4.52 (1.01 to 20.25)*
Difficulty concentrating	1.53 (1.13 to 2.08)**	2.35 (0.80 to 6.88)	3.15 (1.06 to 9.32)*
Unsteadiness	1.71 (1.20 to 2.44)**	6.12 (1.60 to 23.46)**	1.09 (0.32 to 3.76)

*p < 0.05, **p < 0.01, ***p < 0.001)

No allergic responses were noted in this cohort. The most significant biochemical finding was hyponatremia on high dose OXC, 23.4 % of the patients reported low sodium levels i.e. between 134–127 mmol/L in one of their visits; from which 2 out of 3 were on OXC. No low levels of folate, abnormal liver function tests or bone calcium levels were reported. A fall leading to a fracture of the humerus of one elderly Caucasian patient was reported. This was thought to be due to high dose CBZ leading to ataxia. Osteoporosis is unlikely to be due to CBZ as the patient was not on it for a long time.

When treating chronic neuralgiform conditions affecting the face, clinicians frequently have to go above recommended maximum prescription doses of both CBZ and OXC in order to reach therapeutic benefit. This comes at the cost of frequent development of side effects, leaving clinicians without any clear evidence-based guidance. Recent reviews of both general and analgesic drug trials have shown the lack of systematic reporting of adverse effects [10, 1]. For epileptic patients attempts have been made to develop clinically useful standardized methods of reporting adverse effects, which do not involve complex neuropsychological tests such as the Liverpool AEP [5], and the A–B Neuropsychological Assessment Schedule (ABNAS) [11]. A retrospective study by Di Stefano and colleagues [3] reported significant side effects to OXC and CBZ in patients with TN but provided no indication of how these were ascertained. This is the first study that has attempted to use a psychometrically tested self-complete questionnaire to ascertain the magnitude of these side effects in this group of patients. In our study we have shown a relationship between the dose of CBZ and OXC and the total score on a questionnaire that records and rates side effects. Scores associated with toxicity were associated with a lower dose in female patients than in male patients. Differences between CBZ and OXC were clinically modest and not statistically significant.

With the increasing need to ensure equality in clinical trials there is growing awareness that there are specific pharmacological differences not just in drug efficacy but also adverse drug reactions between genders. Numerous reviews have described the genetic, hormonal and psychosocial basis for differences in both pain perception and responses to various analgesics [12]. It is reported that in up to 40 % of drugs there are differences in pharmacokinetics in males and females [13]. Classes of drugs in which this is particularly evident is opioids, anti arrhythmic, HIV related [14], and psychotropic drugs [15].

A variety of hypotheses have been put forward for these differences including pharmacokinetics, women being prescribed more medications, and pharmacodynamics differences [16]. Differences in body weight, percentage body fat and increased vigilance and desire to report side effects are likely to have some effect [13]. However when these are corrected differences remain. Some of these effects could be related to sex hormones but other mechanisms may be involved [14]. The activity of the cytochrome P450 (CYP) and renal excretion show differences [13] and CBZ uses the CYP system whereas OXC uses the renal system so both could have an influence. Our main finding suggests that females report significantly higher side-effects than males and also have toxic level score of  > 45 at significantly lower doses than males and this is not related to body weight. In this study we excluded patients taking psychotropic drugs but not those on hypertensive therapies. Although it is often suggested that adherence to drugs is likely to be better in females in TN the pain severity is so high that adherence is not an issue.

International and national guidelines suggest that the primary drug of choice for patients with TN is CBZ and the second drug of choice is OXC with lamotrigine, baclofen to be used if there is poor tolerability to the primary drug [17] and NICE CG173 suggests the primary drug to be CBZ [18]. Although used extensively as first line drug for TN in the Scandinavian countries, OXC is not approved for this use by FDA in the US and British National Formulary in the UK lists CBZ as the only licensed one. The evidence available suggests that CBZ and OXC are equally effective for pain relief but tolerability is better with OXC [19, 3], our data does not support this statement. A variety of other AEDs have been shown in smaller studies to be effective e.g. gabapentin and pregabalin but there is weak evidence on use of non-AEDs in this condition [20]. There is no evidence for the use of two AEDs in TN [17].

### Range of side-effects associated with AED

Our findings are consistent with those reported previously [21, 3]. Memory and concentration seem to suffer the most [22], Salinsky and colleagues [23] showed that OXC also affects cognitive function. Alteration of behavior, such as changes in mood and cognition, attributed to AEDs are multifaceted and can differ considerably between patients [24]. A recent review has also pointed out that there is a small risk of increased suicide when using AED especially if depression is caused by chronic pain [25] and this clinic uses the Hospital Anxiety and Depression scale to monitor this. Cognitive function is also affected by pain [26] complicating assessment further, warranting baseline measurement before any pharmacotherapy is considered.

AED therapy in general causes changes in calcium metabolism leading to decreased bone mass with the risk of osteoporotic fractures. As elderly population represents a substantial component of our cohort, osteoporosis combined with ataxia and an increased risk of falls is a significant consideration when initiating any AED therapy [27]. Syndrome of inappropriate antidiuretic hormone (SIADH) secretion and the associated hyponatraemia has been associated with some AEDs, including CBZ and OXC. Hyponatraemia has previously been shown to be dose-dependent [28] and was found in this study. Folate deficiencies can occur with the use of CBZ [29] and this has also been monitored routinely. CBZ has also been linked to severe dose-independent reactions such as Stevens–Johnson syndrome or more severe toxic epidermal necrolysis. Carriers of the human leukocyte antigen (HLA) HLA-B*1502, HLA*3101 and HLA*1511 alleles, especially of Asian ancestry, demonstrate a predisposition to developing this adverse reaction. Recently, American Food and Drug Administration (FDA) recommended that before commencing CBZ, all at risk patients should be screened for the relevant genetic risk factors [30].

### Limitations of study

Numbers especially of CBZ users were too few to find significant differences in profile of side effects between CBZ and OXC although Beydoun [9, 19] reporting their RCT comparing both drugs suggested there were significant differences with CBZ patients reporting a higher frequency of vertigo, fatigue and dizziness. Validity of AEP questionnaire may be affected by direct effect of pain on side effects included in the questionnaire as the tool has not previously been used in patients with chronic pain. Even though Tan and colleagues [31] have pointed out that making patients aware of side effects increases their propensity to report them it remains important to establish how significant the side effects are as this can prove to be an important indicator that a change in management needs to be considered. With increasingly short appointment times it is impossible to go through each of the adverse effects in this questionnaire but it is relatively easy to discuss a completed questionnaire.

Prospective observational explorative studies have clearly defined limitations [32, 33]. On the other hand, one of the major limitations of RCTs in the context of tolerability is frequently unaccounted rate of drop-outs in the treatment arm due to side-effects and intolerance, skewing the outcome towards positive results based on the efficacy of the drug in question alone. Our cohort reflects clinicians experience and while adhering to national and international guidelines, offers additional information on management of patients on doses exceeding the recommended range, when to expect toxicity and encourages AED rotation in presence of toxicity development. The results also reflect the patient experience which showed that tiredness and cognitive impairment were the major problems [4].

Even though usage of standardized questionnaires such as the Liverpool AEP adds a layer of data reported by the patients, it is associated with its own limitations. Over reporting due to unclear instructions, general feeling ‘on the day’, over-generalisation, desire to go onto a different medication, and drug-drug interaction are to name a few confounders which may affect the quality of data collected [34]. In our particular study, one of the limitations was that interpretation of some of the questions proved to be challenging and some participants would for instance circle ‘oral problem’ when in fact they would experience pain in the area. One of the suggestions with the aim of overcoming this and related limitations is a potential use of the other scale A–B Neuropsychological Assessment Schedule (ABNAS), which seems to be more explicit; however toxicity level correlate has not been established [35]. Discussion of the results reported by any questionnaire, however, aids shared decision-making when it comes to considering drug rotation, surgical treatment options or seeking input from the pain management program.

Zakrzewska and Patsalos [28] showed side effects were a major reason for changing from CBZ to OXC rather than efficacy which has been shown to be equal [9, 19], but no formal measure was used - patients just reported them on a pain diary. In epilepsy, Gilliam and colleagues [8] suggested that a change to an AED with a lower adverse event burden may improve quality of life in patients with epilepsy without sacrificing seizure control.

### Future research

For future research it would be necessary to recruit larger numbers of patients on both CBZ and OXC and other AEDs such as lamotrigine, gabapentin and pregablin in order to compare better tolerability especially aspects of cognitive dysfunction in order to provide clinicians with the better guidance on how to troubleshoot drug intolerance. There are more objective methods using sophisticated computer programs such as Kinematic Assessment Tool (KAT) to assess these effects. The KAT is a series of touch screen computerised tasks, which have already been evidenced as providing objective measures of cognitive and motoric performance in adults and children without neurological impairment [36]. It is also feasible to look into the experience of patients on multiple medications rather than on a single agent as combination therapy may not always result in a higher number of side effects.

## Conclusions

When adverse effects are measured using a psychometrically tested questionnaire both OXC and CBZ demonstrate that as dosages increase significant cognitive side effects are noted. These side effects are significantly different in males and females and this suggests that females may be more at risk from side effects than males.
